# A new species of the *Hebius atemporalis* complex (Squamata, Natricidae) from southeastern Yunnan, China

**DOI:** 10.3897/zookeys.1289.189681

**Published:** 2026-08-11

**Authors:** Shuo Liu, JiShan Wang, Mian Hou, Tao Deng, Chaowu Zhang, Mo Wang, Dingqi Rao

**Affiliations:** 1 Kunming Natural History Museum of Zoology, Kunming Institute of Zoology, Chinese Academy of Sciences, Kunming, Yunnan 650223, China Yunnan Academy of Biodiversity, Southwest Forestry University Kunming China; 2 Key Laboratory of National Forestry and Grassland Administration on Biodiversity Conservation on the Qinghai-Xizang Plateau, Kunming Institute of Zoology, CAS, Kunming, Yunnan 650201, China Key Laboratory of National Forestry and Grassland Administration on Biodiversity Conservation on the Qinghai-Xizang Plateau, Kunming Institute of Zoology, CAS Kunming China; 3 Yunnan Academy of Biodiversity, Southwest Forestry University, Kunming, Yunnan 650224, China Kunming Natural History Museum of Zoology, Kunming Institute of Zoology, Chinese Academy of Sciences Kunming China; 4 Southwest Survey and Planning Institute of National Forestry and Grassland Administration, Kunming, Yunnan 650031, China Kunming Institute of Zoology, Chinese Academy of Sciences Kunming China; 5 College of Continuing (Online) Education, Sichuan Normal University, Chengdu, Sichuan 610066, China College of Continuing (Online) Education, Sichuan Normal University Chengdu China; 6 Maguan County Management and Protection Branch of Laojunshan Provincial Nature Reserve, Maguan, Yunnan 663700, China Southwest Survey and Planning Institute of National Forestry and Grassland Administration Kunming China; 7 Kunming Institute of Zoology, Chinese Academy of Sciences, Kunming, Yunnan 650201, China Maguan County Management and Protection Branch of Laojunshan Provincial Nature Reserve Maguan China

**Keywords:** Complex, cytb, keelback snake, morphology, taxonomy, Wenshan Prefecture

## Abstract

A new species of the *Hebius atemporalis* complex is described from Wenshan Prefecture, Yunnan Province, China, based on morphological and molecular evidence. The new species can be distinguished from other species of the complex by the differences in the ratio of tail length to total length, the number of ventral and subcaudal scales, the number of maxillary teeth, and the coloration of the iris and anterior body. Phylogenetically, the new species is separated from other species of the complex by an uncorrected p-distance of 6.0–7.8% in the mitochondrial cytochrome *b* gene. This study further highlights the cryptic diversity in the *H. atemporalis* complex and the need to re-examine previous records of *H. atemporalis* and specimens previously considered *H. atemporalis* from central-southern China, as well as northeastern Vietnam.

## Introduction

*Hebius* Thompson, 1913, commonly known as keelback snake, is a genus with a very rich diversity of species whose species diversity is seriously underestimated (Liu et al. 2025; Le et al. 2026; Li et al. 2026). This genus currently represents the most diverse lineage of natricid snakes. Species are usually small to medium sized, semi-aquatic, non-venomous snakes, widely distributed across East and Southeast Asia (e.g., Guo et al. 2014; Liu et al. 2018; Purkayastha and David 2019; Deepak et al. 2022; Hauser et al. 2022; Ren et al. 2022; Ma et al. 2023; Xu et al. 2023, 2024; Bohra et al. 2025; Nguyen et al. 2025; Uetz et al. 2026).

The *Hebius atemporalis* complex is widely distributed across northern Vietnam and southern China and characterized by the absence of temporal scales or presence of only small narrow temporal scales enclosed by postoculars, 17-17-17 dorsal scale rows, a white ventral head surface with scattered black spots or streaks, strongly black-edged infralabial sutures, and indistinct dorsolateral stripes composed of a series of small light spots (Zhao et al. 1998; Zhao 2006; Nguyen et al. 2009; Wallach et al. 2014; Nguyen and Poyarkov 2020; Li et al. 2023, 2026). Recently, Li et al. (2026) presented an integrative taxonomic reassessment of the *H. atemporalis* complex, covering specimens from Vietnam and Liupanshui City and Libo County of Guizhou Province, Chenzhou City of Hunan Province, Youyang County of Chongqing Municipality, Yichang City of Hubei Province, Shixing County of Guangdong Province, and Honghe Prefecture and Kunming City of Yunnan Province, China, and described a new species, *H. malnatei* Li, Nguyen, Huang, Poyarkov, David, Li & Ren, 2026. However, Li et al. (2026) did not include any samples from Wenshan Prefecture of Yunnan Province in their study.

During our recent field survey in southern Yunnan Province, China, three snakes of the genus *Hebius* were discovered from Wenshan Prefecture. Combining morphological comparison and phylogenetic analysis, the results indicate that they belong to an unnamed species in the *H. atemporalis* complex. Therefore, we describe a new species of *Hebius* below.

## Materials and methods

One specimen (KIZ2025058) was collected by hand; after being photographed, it was euthanized. Another two specimens were found dead on the road: one (KIZ2025175) intact with only minor damage, and the other (KIZ2025050) squashed by passing vehicles and already dried. All specimens were preserved in approximately 70% ethanol and deposited at Kunming Natural History Museum of Zoology, Kunming Institute of Zoology, Chinese Academy of Sciences (**KIZ**), Kunming, China.

Gene sequences were generated for the three newly collected specimens. A partial fragment of the mitochondrial cytochrome *b* gene (cytb) was amplified and sequenced using the primers L14910 and H16064 (Burbrink et al. 2000). The molecular experiment was completed by Sangon Biotech Co., Ltd. *Herpetoreas burbrinki* Guo, Zhu, Liu, Zhang, Li, Huang & Pyron, 2014 and *H. tpser* Ren, Jiang, Huang, David & Li, 2022 were used as out groups according to Li et al. (2026). The sequences of congeners and outgroups were obtained from GenBank (Table 1).

**Table 1.** Voucher and GenBank accession numbers and localities of the samples of *Hebius* and outgroup taxa used in this study.

**Species**	**Voucher**	**Locality**	**Accession**	**Reference**
*Hebius yangi* sp. nov.	KIZ2025058	Malipo, Yunnan, China	PZ753911	This study
*Hebius yangi* sp. nov.	KIZ2025175	Malipo, Yunnan, China	PZ753909	This study
*Hebius yangi* sp. nov.	KIZ2025050	Malipo, Yunnan, China	PZ753910	This study
*Hebius andreae*	VNUF R.2017.25	Khammouane, Laos	MK253674	Ziegler et al. 2019
*Hebius annamensis*	FMNH 258637	Kaleum, Xekong, Laos	OK315812	Deepak et al. 2022
*Hebius atemporalis*	ZMMU NAP-07877	Pu Hoat, Nghe An, Vietnam	OK315813	Deepak et al. 2022
*Hebius atemporalis*	ZMMU NAP-09038	Pu Hoat, Nghe An, Vietnam	PX686993	Li et al. 2026
*Hebius cf. atemporalis*	GP1626	Shixing, Guangdong, China	KJ685680	Guo et al. 2014
*Hebius cf. atemporalis*	CIB 121808	Libo, Guizhou, China	PX686994	Li et al. 2026
*Hebius bitaeniatus*	AUP-00062	Chiang Mai, Thailand	OK315816	Deepak et al. 2022
*Hebius boulengeri*	GP1789	Guangdong, China	KJ685684	Guo et al. 2014
*Hebius chapaensis*	VNMN 06102	Lao Cai, Vietnam	MH778702	Ren et al. 2018
*Hebius citrinoventer*	ANU20230016	Yingjiang, Yunnan, China	PP472750	Xu et al. 2024
*Hebius clerki*	CAS 215036	Yunnan, China	KJ685666	Guo et al. 2014
*Hebius concelarus*	KUZ R20253	Miyakojimashi, Japan	AB989268	Kaito and Toda 2016
*Hebius craspedogaster*	CIB 119635	Enshi, Hubei, China	OR361322	Li et al. 2023
*Hebius deschauenseei*	AUP-00182	Chiang Mai, Thailand	OK315827	Deepak et al. 2022
*Hebius gilhodesi*	CAS 221504	Kachin, Myanmar	KJ685668	Guo et al. 2014
*Hebius igneus*	AMNH 148575	Tuyen Giang, Vietnam	KJ685665	Guo et al. 2014
*Hebius ishigakiensis*	KUZ R33044	Iriomotejima, Japan	AB989294	Kaito and Toda 2016
*Hebius jingdongensis*	CIB 119044	Jingdong, Yunnan, China	OR285310	Ma et al. 2023
*Hebius johannis*	KIZ 014484	Yunnan, China	MZ570479	Hou et al. 2021
*Hebius khasiensis*	CAS 221525	Kachin, Myanmar	KJ685669	Guo et al. 2014
*Hebius malnatei*	CIB 122550	Liupanshui, Guizhou, China	PX686990	Li et al. 2026
*Hebius malnatei*	CIB 122551	Liupanshui, Guizhou, China	PX686991	Li et al. 2026
*Hebius malnatei*	CIB 122553	Mengzi, Yunnan, China	PX686989	Li et al. 2026
*Hebius malnatei*	CIB 122554	Mengzi, Yunnan, China	PX686988	Li et al. 2026
*Hebius malnatei*	CIB 122556	Kunming, Yunnan, China	PX686995	Li et al. 2026
*Hebius malnatei*	CIB 122558	Kunming, Yunnan, China	PX686992	Li et al. 2026
*Hebius malnatei*	HS11001	Mengzi, Yunnan, China	MK201299	Li et al. 2020
*Hebius malnatei*	HT550	China	LC325320	Deepak et al. 2022
*Hebius maximus*	CIB118635	Yibin, Sichuan, China	OP937185	Li et al. 2022
*Hebius metusia*	GP1712	Sichuan, China	KJ685682	Guo et al. 2014
*Hebius modestus*	KIZ037715	Tengchong, Yunnan, China	MZ570481	Hou et al. 2021
*Hebius ngoclinhensis*	ILS H.20386	Ngoc Linh, Kon Tum, Vietnam	PX584656	Le et al. 2026
*Hebius octolineatus*	KIZ026445	Kunming, Yunnan, China	MZ570484	Hou et al. 2021
*Hebius optatus*	GP1885	Guizhou, China	KJ685687	Guo et al. 2014
*Hebius popei*	GP2169	Hainan, China	KJ685692	Guo et al. 2014
*Hebius pryeri*	KUZ R67983	Amamioshima, Japan	AB989102	Kaito and Toda 2016
*Hebius sangzhiensis*	SYNU 08070350	Sangzhi, Hunan, China	MK340763	Zhou et al. 2019
*Hebius sauteri*	CIB 118516	Guangdong, China	OP937178	Li et al. 2022
*Hebius septemlineatus*	KIZ 037706	Tengchong, Yunnan, China	MZ570485	Hou et al. 2021
*Hebius shantianfangi*	KIZ 064970	Mengla, Yunnan, China	PX206432	Liu et al. 2025
*Hebius taronensis*	CAS 224426	Myanmar	OK315828	Deepak et al. 2022
*Hebius venningi*	CAS 233206	Kachin, Myanmar	KJ685670	Guo et al. 2014
*Hebius vibakari*	KUZ R21587	Kyoto, Japan	AB989302	Kaito and Toda 2016
*Hebius vogeli*	AMNH 147155	Tam Dao, Phu Tho, Vietnam	KJ685662	Nguyen et al. 2025
*Hebius weixiensis*	KIZ 035740	Weixi, Yunnan, China	MZ570488	Hou et al. 2021
*Hebius yanbianensis*	GP4006	Yanbian, Sichuan, China	MH532291	Liu et al. 2018
*Hebius youjiangensis*	ANU20220010	Baise, Guangxi, China	OQ085073	Xu et al. 2023
*Herpetoreas tpser*	CIB 107855	Xizang, China	OM313292	Ren et al. 2022
*Herpetoreas burbrinki*	YBU 071128	Xizang, China	GQ281781	Guo et al. 2014

The alignment of sequences and the calculation of the uncorrected pairwise distances were conducted in MEGA 11 (Tamura et al. 2021). The best-fit substitution models, GTR+F+I+G4 for Bayesian inference and TIM2+F+R4 for maximum likelihood analysis, respectively, were selected using the Bayesian information criterion in ModelFinder (Kalyaanamoorthy et al. 2017). Bayesian inference was performed in MrBayes 3.2.6 (Ronquist et al. 2012) and the Markov chains were run for 2,000,000 generations and sampled every 100 generations, with the first 25% of the trees discarded as burn-in. Maximum likelihood analysis was performed in IQ-TREE 1.6.12 (Nguyen et al. 2015) and the nodal support was estimated by 1000 ultrafast bootstrap replicates.

Morphometric measurements were recorded for the two intact specimens. Methodology of definitions followed Li et al. (2026): SVL, snout-vent length; TaL, tail length; TL, total length; HL, head length; HW, head width; PrO, preocular; PtO, postocular; L, loreal; SL, supralabial; SL-E, supralabials entering orbit; IL, infralabial; AT, anterior temporal; DSR, dorsal scale rows; VEN, ventral; SC, subcaudal; and MT, maxillary teeth. The sex of specimens was determined by examining whether the hemipenes were present.

Morphological comparisons were based on original and additional species descriptions (Maki 1931; Malnate 1962; Zhao et al. 1998; David et al. 2013, 2015, 2021, 2023; Ren et al. 2018; Zhou et al. 2019; Hou et al. 2021; Hauser et al. 2022; Li et al. 2022, 2026; Ma et al. 2023; Xu et al. 2023, 2024; Gao et al. 2024; Bohra et al. 2025; Liu et al. 2025; Nguyen et al. 2025; Le et al. 2026).

## Results

**Figure 1.**
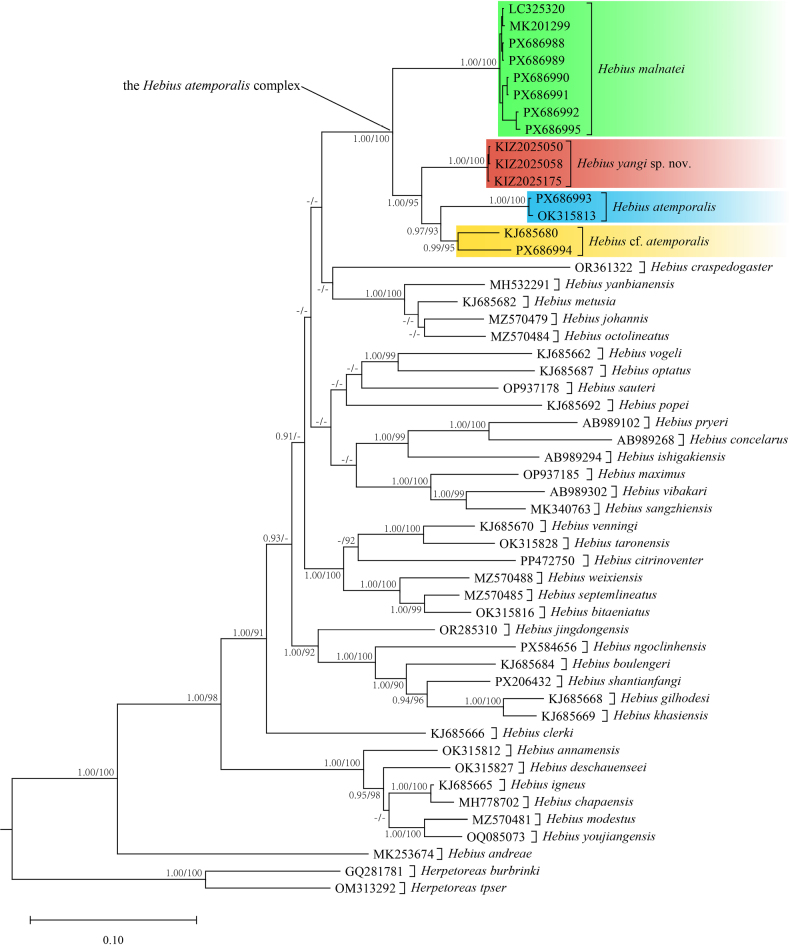
Bayesian phylogenetic tree of the genus *Hebius* based on cytb sequences. The numbers before and behind “/” indicate the Bayesian posterior probabilities (≥ 0.90 retained) and maximum likelihood ultrafast bootstrap values (≥ 90 retained), respectively.

The amplified cytb fragment and the final alignment are 1106 bp and 1108 bp in length, respectively. Bayesian inference and maximum likelihood analyses yielded essentially consistent topologies (Fig. 1). The newly collected specimens from Wenshan Prefecture were recovered as a distinct lineage in the *Hebius atemporalis* complex with strong support (Bayesian posterior probability 1.00 and maximum likelihood bootstrap support 95). The uncorrected pairwise distances between the sequences of the newly collected specimens from Wenshan Prefecture and the sequences of *H. atemporalis* sensu stricto from northern Vietnam, *H. cf. atemporalis* from central-southern China, and *H. malnatei* from southwestern China were 7.6%, 6.0%, and 7.8%, respectively (Suppl. material 1: table SS1).

### Hebius yangi sp. nov.

Taxon classificationAnimaliaSquamataColubridae

D763557D-F326-50DE-A439-0F1FC35C578D

https://zoobank.org/359C306B-4246-45CF-A35A-D534919D0412

Figs 2, 3, 4

#### Type material.

***Holotype***. KIZ2025058, adult male, collected on 3 May 2025 by local villagers from Xiajinchang Township, Malipo County, Wenshan Prefecture, Yunnan Province, China (23°11'7"N, 104°48'12"E; 1450 m a.s.l.). ***Paratype***. KIZ2025175, adult male, collected on 2 November 2025 by local villagers from the same locality as the holotype.

**Figure 2.**
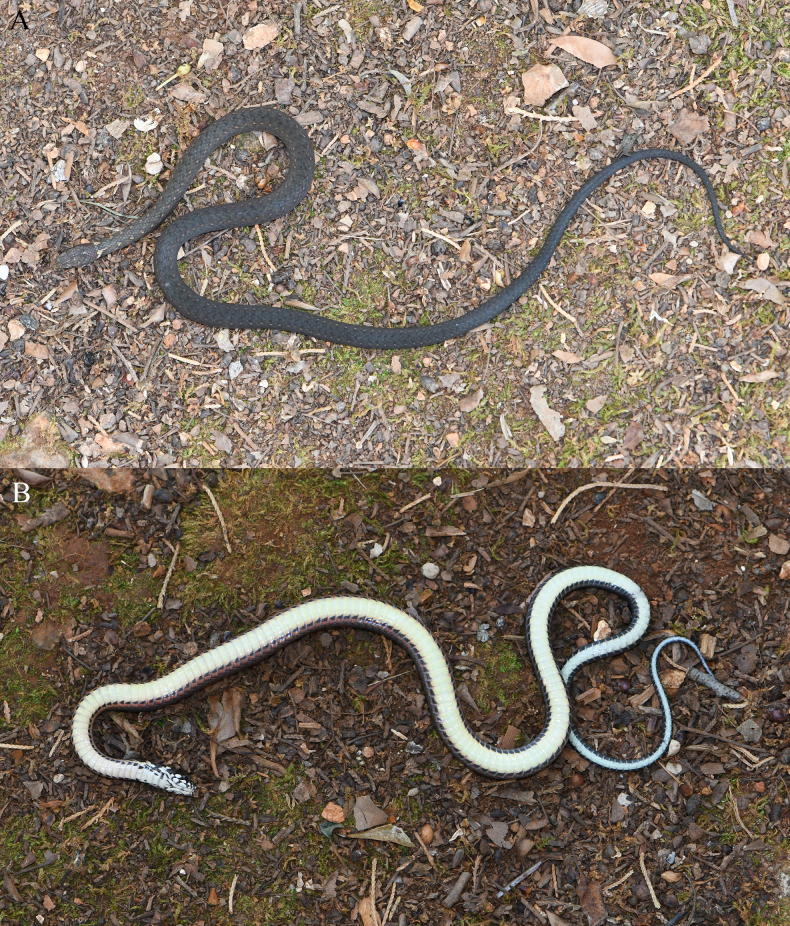
The holotype (KIZ2025058) of *Hebius yangi* sp. nov. in life. **A**. Dorsal view; **B**. Ventral view.

**Figure 3.**
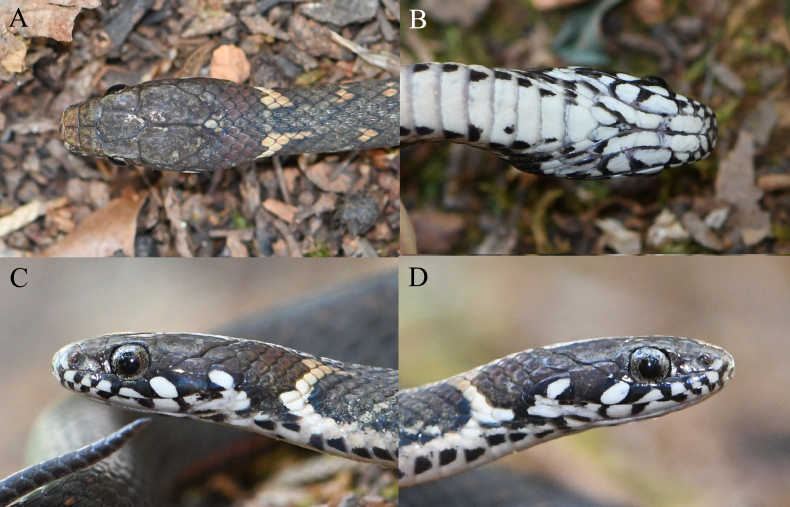
Close-up views of the head of the holotype (KIZ2025058) of *Hebius yangi* sp. nov. in life. **A**. Dorsal view; **B**. Ventral view; **C**. Left view; **D**. Right view.

**Figure 4.**
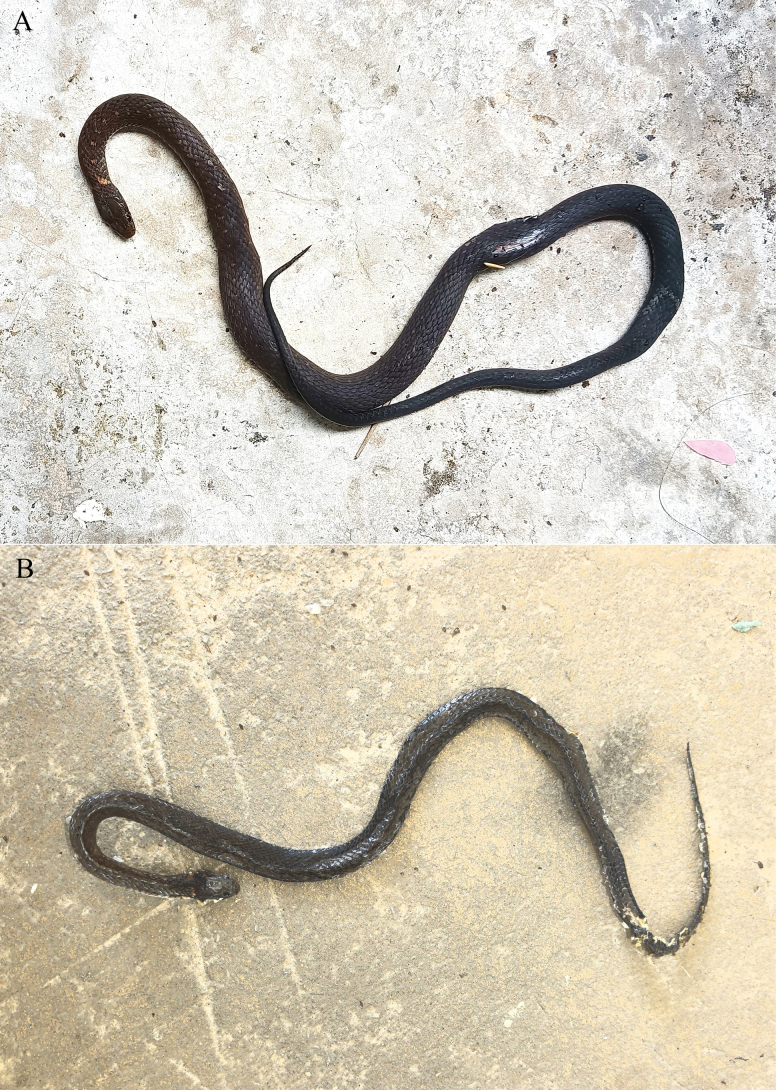
The two road-kills of *Hebius yangi* sp. nov. when they were discovered. **A**. Paratype KIZ2025175; **B**. Specimen KIZ2025050.

#### Additional specimen.

KIZ2025050, adult male, collected on 24 April 2025 by local villagers from the same locality as the holotype.

#### Diagnosis.

Body size relatively small, SVL 237–306 mm; tail moderately long, TaL/TL 0.32–0.33; dorsal scale rows 17-17-17, all keeled except the outermost rows; ventrals 130–131; subcaudals 80–85, paired; supralabials six, third and fourth entering orbit; infralabials seven; maxillary teeth 32–34, gradually enlarged posteriorly; dorsal surface grayish brown to blackish brown with a series of neatly arranged small orange spots on dorsolateral body on each side; ventral surface of head white, scattered with black streaks on scale sutures; iris brownish gray; lip region black and white alternately; neck collar present, discontinuous.

#### Etymology.

The specific epithet is in commemoration of the late renowned Chinese herpetological taxonomist Prof. Dr Datong Yang. We suggest “Yang’s Keelback” as the common English name and “杨氏腹链蛇” as the common Chinese name.

#### Description of holotype.

Relatively small in size, SVL 237 mm, TL 355 mm; body slender, tail moderately long, TaL/TL 0.33; head narrow and elongate, HL/HW 1.92, approximately trapezoidal, moderately distinct from neck; snout relatively short, length 1.5 times of horizontal diameter of eye, narrowing anteriorly, tip blunt in dorsal view and rounded in lateral view; canthus rostralis distinct; eyes relatively large, ratio of horizontal diameter of eye to head length 0.19, pupil round.

Rostral approximately rectangular, width 2.1 times of height, slightly visible from above; nasal large, polygonal, longer than high, divided into anterior and posterior sections, bordered by first two supralabials, rostral, internasal, and prefrontal; internasals two, approximately trapezoidal; prefrontals two, larger than internasals, visible in lateral view; frontal shield shaped, length 1.6 times of width; parietals large, elongate, medial suture length equal to length of frontal; one supraocular on each side, longer than wide; one loreal on each side, approximately square, not entering orbit; one preocular on each side, vertically elongate; subocular absent; two postoculars on each side, upper one slightly larger than lower one; one anterior temporal on each side, small; two posterior temporals, larger than anterior temporal; fifth supralabial in contact with parietal, anterior temporal not in contact with posterior temporals; six supralabials on each side, third and fourth entering orbit, fifth largest; seven infralabials on each side, first pair in contact with each other, first to fourth in contact with anterior chin shields, fourth and fifth in contact with posterior chin shields; posterior chin shields clearly longer than anterior chin shields.

Dorsal scales in 17-17-17 rows, all keeled except outermost row on each side, scales of outermost row on each side slightly enlarged, apical pits absent; ventrals 130, preceded by two preventrals; cloacal plate divided; subcaudals 85, paired.

Maxillary teeth 34, without diastema, gradually enlarged posteriorly.

#### Coloration of holotype in life.

Dorsal surface of head grayish brown; occipital region purplish brown; neck collar present, light orange, broken in middle, dividing into two disconnected parts on left and right; dorsal surface of body grayish brown anteriorly to blackish brown posteriorly, speckled with indistinct black blotches, a series of neatly arranged small orange spots on dorsolateral body on each side; dorsal surface of tail grayish black. Lateral surface of head brownish black; loreal region grayish white; iris brownish gray; lip region black and white alternately, first to fourth supralabials mostly white with wide black edges, fifth and sixth supralabials brownish black, each with a large black-edged elliptical white spot anteriorly. Ventral surface of head white with wide or narrow black streaks on scale sutures. Ventral surface of body creamy white with a series of black blotches on each side; lateral margins of ventrals light red anteriorly to gray posteriorly. Ventral surface of tail grayish white with black margins on each side.

#### Variation.

Measurements and scalation data for the two type specimens and the one additional specimen are summarized in Table 2. Both the other two specimens are very similar to the holotype in morphological characteristics and coloration, except that the fifth supralabial scale is not in contact with the parietal scale but separated by a temporal scale on each side, and the series of orange spots on the dorsolateral body is more indistinct in the paratype.

**Table 2.** Measurements (in mm) and scalation characteristics of *Hebius yangi* sp. nov. N/A = not available.

	**KIZ2025058 Holotype** ♂	**KIZ2025175 Paratype** ♂	**KIZ2025050** ♂
SVL	237	306	248
TaL	118	143	116
TL	355	449	364
HL	11.7	14.1	N/A
HW	6.1	7.8	N/A
PrO	1/1	1/1	N/A
PtO	2/2	2/2	N/A
L	1/1	1/1	N/A
SL	6/6	6/6	N/A
SL-E	3–4/3–4	3–4/3–4	N/A
IL	7/7	7/7	N/A
AT	1/1	1/1	N/A
DSR	17-17-17	17-17-17	17-17-17
VEN	130	130	131
SC	85	80	82
MT	34	32	N/A

#### Comparisons.

*Hebius yangi* sp. nov. differs from *H. andreae* (Ziegler & Le, 2006), *H. bitaeniatus* (Wall, 1925), *H. boulengeri* (Gressitt, 1937), *H. celebicus* (Peters & Doria, 1878), *H. clerki* (Wall, 1925), *H. concelarus* (Malnate, 1963), *H. craspedogaster* (Boulenger, 1899), *H. deschauenseei* (Taylor, 1934), *H. flavifrons* (Boulenger, 1887), *H. gilhodesi* (Wall, 1925), *H. groundwateri* (Smith, 1922), *H. igneus* David, Vogel, Nguyen, Orlov, Pauwels, Teynié & Ziegler, 2021, *H. inas* (Laidlaw, 1901), *H. ishigakiensis* (Malnate & Munsterman, 1960), *H. jingdongensis* Ma, Shi, Ayi & Jiang, 2023, *H. johannis* (Boulenger, 1908), *H. kerinciensis* (David & Das, 2003), *H. khasiensis* (Boulenger, 1890), *H. lacrima* Purkayastha & David, 2019, *H. leucomystax* (David, Bain, Nguyen, Orlov, Vogel, Vu & Ziegler, 2007), *H. metusia* (Inger, Zhao, Shaffer & Wu, 1990), *H. miyajimae* (Maki, 1931), *H. modestus* (Günther, 1875), *H. ngoclinhensis* Le, Phan, Nguyen, Murphy, Lam, Nguyen & Che, 2026, *H. octolineatus* (Boulenger, 1904), *H. optatus* (Hu & Zhao, 1966), *H. parallelus* (Boulenger, 1890), *H. petersii* (Boulenger, 1893), *H. popei* (Schmidt, 1925), *H. pryeri* (Boulenger, 1887), *H. sanguineus* (Smedley, 1932), *H. sangzhiensis* Zhou, Qi, Lu, Lyu & Li, 2019, *H. sarasinorum* (Boulenger, 1896), *H. septemlineatus* (Schmidt, 1925), *H. shantianfangi* Liu, Wang, Hou, Zhang, Wang, Zong, Zhou, Rao, David & Vogel, 2025, *H. terrakarenorum* Hauser, Smits & David, 2022, *H. vibakari* (Boie, 1826), *H. viperinus* (Schenkel, 1901), *H. vogeli* Nguyen, Li, Huang, Han, Nguyen, Poyarkov, David & Ren, 2025, *H. weixiensis* Hou, Yuan, Wei, Zhao, Liu, Wu, Shen, Chen, Guo & Che, 2021, and *H. yanbianensis* Liu, Zhong, Wang, Liu & Guo, 2018 by having 17 dorsal scale rows at midbody (vs 19 or 15).

*Hebius yangi* sp. nov. differs from *H. annamensis* (Bourret, 1934), *H. chapaensis* (Bourret, 1934), *H. nigriventer* (Wall, 1925), *H. taronensis* (Smith, 1940), *H. venningi* (Wall, 1910), and *H. youjiangensis* Yang, Xu, Wu, Gong, Huang & Huang, 2023 by having a white venter (vs dark venter).

*Hebius yangi* sp. nov. differs from *H. arquus* (David & Vogel, 2010) and *H. citrinoventer* Xu, Yang, Gong, Ouyang, Weng, Deng, Huang & Peng, 2024 by having fewer ventrals (VEN ≤ 131 vs ≥ 146).

*Hebius yangi* sp. nov. differs from *H. frenatus* (Dunn, 1923) and *H. sarawacensis* (Günther, 1872) by having six supralabials (vs eight).

*Hebius yangi* sp. nov. differs from *H. maximus* (Malnate, 1962) and *H. sauteri* (Boulenger, 1909) by having more maxillary teeth (MT 32–34 vs 23–27).

*Hebius yangi* sp. nov. differs from *H. atemporalis* sensu stricto from northern Vietnam by having more subcaudal scales (SC 80–85 vs 63–79), more ventrals plus subcaudals (VEN+SC 210–215 vs 198–209), seven infralabials (vs six, rarely seven), one row of smooth dorsal scales on each side on anterior body (vs three to six rows), more maxillary teeth (MT 32–34 vs 27–30), a brownish gray iris (vs copper colored), a brown anterior body (vs brownish red), and a discontinuous neck collar (vs continuous) (Table 3).

**Table 3.** Comparison of morphological characteristics between species of the *Hebius atemporalis* complex. Data for *H. atemporalis*, *H. malnatei*, and *H. cf. atemporalis* were obtained from Li et al. (2026).

	***Hebius yangi* sp. nov**.	***Hebius atemporalis***	***Hebius malnatei***	***Hebius cf. atemporalis***
Max SVL	306 mm (*N* = 3)	292 mm (*N* = 11)	416 mm (*N* = 12)	294 mm (*N* = 4)
TaL/TL	0.32–0.33 (*N* = 3)	0.30–0.33 (*N* = 7)	0.22–0.27 (*N* = 10)	0.29–0.30 (*N* = 2)
VEN	130–131 (*N* = 3)	123–136 (*N* = 11)	146–156 (*N* = 12)	132–140 (*N* = 4)
SC	80–85 (*N* = 3)	63–79 (*N* = 8)	60–70 (*N* = 10)	69–74 (*N* = 3)
VEN+SC	210–215 (*N* = 3)	198–209 (*N* = 8)	211–223 (*N* = 10)	203–206 (*N* = 3)
DSR	17-17-17	17-17-17	17-17-17	17-17-17
SL	6	6	6 (rarely 5)	6
SL-E	3–4	3–4	3–4 (rarely 3)	3–4
IL	7	6 (rarely 7)	7 (rarely 6)	6 or 7
PrO	1	1	1	1
PtO	2	2	2	2
AT	1	0 or 1	0 or 1	1
MT	32–34	27–30	26–29	32–33
Anterior body color	Brown	Brownish red	Brown	Brownish red
Neck collar	Discontinuous	Continuous	Absent	Discontinuous

*Hebius yangi* sp. nov. differs from *H. malnatei* by having smaller maximum SVL (Max SVL 306 mm vs 416 mm), a relatively longer tail (TaL/TL 0.32–0.33 vs 0.22–0.27), fewer ventrals (VEN 130–131 vs 146–156), more subcaudals (SC 80–85 vs 60–70), one row of smooth dorsal scales on each side on anterior body (vs six rows), more maxillary teeth (MT 32–34 vs 26–29), brownish gray iris (vs copper colored), and a light orange neck collar (vs no neck collar) (Table 3).

*Hebius yangi* sp. nov. differs from *H. cf. atemporalis* from southern China by having a relatively longer tail (TaL/TL 0.32–0.33 vs 0.29–0.30), fewer ventrals (VEN 130–131 vs 132–140), more subcaudals (SC 80–85 vs 69–74), more ventrals plus subcaudals (VEN+SC 210–215 vs 203–206), one row of smooth dorsal scales on each side on anterior body (vs three to six rows), brownish gray iris (vs copper colored), and a brown anterior body (vs brownish red) (Table 3).

#### Distribution.

**Figure 5.**
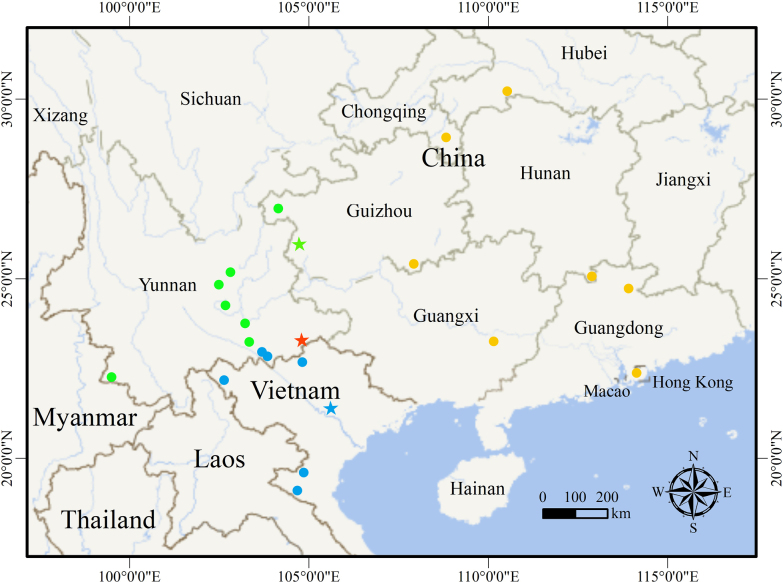
Map showing distributions of the *Hebius atemporalis* complex. Red: *Hebius yangi* sp. nov. (this study), blue: *H. atemporalis* sensu stricto (Nguyen et al. 2009; Wallach et al. 2014; Nguyen and Poyarkov 2020; Li et al. 2026; this study), green: *H. malnatei* (Li et al. 2026; this study), and yellow: *H. cf. atemporalis* (Zhao et al. 1998; Zhao 2006; Li et al. 2023, 2026). Star indicates type locality.

The new species is currently known only from Malipo County, Wenshan Prefecture, Yunnan Province, China (Fig. 5).

#### Ecology notes.

One specimen was collected while it was traversing the road during the day, and the other two specimens were found dead on the road. There are evergreen broad-leaved forests and farmland on the sides of the road, and there is no water body nearby. More ecological information about this species is unknown.

## Discussion

Li et al. (2026) conducted a comprehensive taxonomic revision of the *Hebius atemporalis* complex, considered the populations from northern Vietnam and central-southern China as *H. atemporalis* sensu stricto, and described the populations from central-eastern Yunnan Province and western Guizhou Province of China as *H. malnatei*. Although they acknowledged the significant genetic divergence and subtle morphological differences between the populations from central-southern China and those from northern Vietnam, they adopted a conservative approach and treated them as a single species. However, based on the genetic divergence and morphological differences (Table 3), we consider that the populations from central-southern China do not belong to *H. atemporalis* sensu stricto, but represent one or multiple distinct species, which we tentatively refer to as *H. cf. atemporalis*. In addition, Yang and Rao (2008) recorded three specimens identified as *H. atemporalis* in Yunnan Province, which were collected from Menglian County of Pu’er City and Hekou and Pingbian counties of Honghe Prefecture, respectively. We re-checked these three specimens and we consider that the specimen from Menglian (KIZ 75I352) belongs to *H. malnatei* as it has 148 ventrals, no anterior temporal, 27–28 maxillary teeth, and no neck collar, while the two specimens from Hekou (KIZ 77I0072) and Pingbian (KIZ 79013) belong to *H. atemporalis* sensu stricto as they have 129–133 ventrals, one anterior temporal, 29–30 maxillary teeth, and distinct neck collar. Therefore, according to current data, *H. atemporalis* sensu stricto is distributed in northern Vietnam and the southernmost Honghe Prefecture in Yunnan Province of China, *H. malnatei* is distributed in central-eastern and southwestern Yunnan Province and western Guizhou Province of China, and *Hebius yangi* sp. nov. is distributed in southeastern Yunnan Province of China. The taxonomic status of the populations previously regarded as *H. atemporalis* from other areas needs further study.

Species of the genus *Hebius* are typically aquatic or semi-aquatic, and they usually feed on fish, frogs, or tadpoles (Zhao et al. 1998; Zhao 2006), however, the species of the *H. atemporalis* complex seem to be more terrestrial. Li et al. (2026) recorded that *H. malnatei* inhabits forests, shrubland, and meadows and feeds on earthworms. In addition, an individual of *H. malnatei* was found in a residential area without ponds or streams nearby from Kunming City, Yunnan Province, China, and an individual referable to the *H. atemporalis* complex from Pu Mat National Park, Nghe An Province, Vietnam, was observed traversing a road in an area distant from water sources (personal communications). Similarly, the specimens of *Hebius yangi* sp. nov. were also discovered in an area without water bodies. Although we have not yet observed this species preying, we speculate that it also feeds on earthworms. We have found a large number of earthworms in the habitat of *Hebius yangi* sp. nov., which partially supports this speculation. The ecological information of this new species is still very scarce and requires more observation.

The population status of *Hebius yangi* sp. nov. is currently unknown. It appears to be very common in its discovery locality, as local villagers report frequently encountering it there. However, this species has hardly been found elsewhere. Perhaps this species is often concentrated in some suitable habitats with high population density. But this does not mean that the overall population density of the species is high. Although there are large forests around the type locality of this species, there are also farmlands nearby. Agricultural production activities and the use of pesticides undoubtedly have a certain impact on this species. Therefore, further investigation is needed to assess the conservation status of this species.

So far, 23 species of the genus *Hebius* have been recorded in Yunnan Province, namely *H. atemporalis*, *H. bitaeniatus*, *H. boulengeri*, *H. chapaensis*, *H. citrinoventer*, *H. clerki*, *H. craspedogaster*, *H. igneus*, *H. jingdongensis*, *H. johannis*, *H. khasiensis*, *H. malnatei*, *H. modestus*, *H. nigriventer*, *H. octolineatus*, *H. optatus*, *H. popei*, *H. sauteri*, *H. septemlineatus*, *H. shantianfangi*, *H. taronensis*, *H. weixiensis*, and *H. yanbianensis* (Wang et al. 2022; Guo and Che 2024; Jiang et al. 2025; Uetz et al. 2026). This study brings the total number of species of *Hebius* in Yunnan Province to 24. However, the actual number of species of this genus distributed in Yunnan may far exceed this, and the diversity is still severely underestimated. More field surveys and further studies on taxonomic issues within the genus will lead to the discovery of more new species.

## Supplementary Material

XML Treatment for Hebius yangi

## Additional information

### Artificial Intelligence (AI) use

The authors accept full responsibility for the content of the manuscript, including the disclosure of any use of AI.

No AI tools were used in the preparation of this manuscript.

### Author ORCIDs

S. Liu https://orcid.org/0000-0001-7825-3006

J. Wang https://orcid.org/0000-0003-4660-163X

M. Hou https://orcid.org/0000-0002-3322-6570

T. Deng https://orcid.org/0009-0006-2420-3533

M. Wang https://orcid.org/0000-0003-3630-8704

D. Rao https://orcid.org/0000-0003-2680-1503

### Data availability

All of the data that support the findings of this study are available in the main text or Supplementary Information.

## Supplementary materials

Supplementary material 1 Supplementary table Data type xls Explanation note Uncorrected pairwise distances (%) between species of *Hebius* based on cytb sequence. https://binary.pensoft.net/file/1742142This dataset is made available under the Open Database License (http://opendatacommons.org/licenses/odbl/1.0/). The Open Database License (ODbL) is a license agreement intended to allow users to freely share, modify, and use this Dataset while maintaining this same freedom for others, provided that the original source and author(s) are credited. Shuo Liu, JiShan Wang, Mian Hou, Tao Deng, Chaowu Zhang, Mo Wang, Dingqi Rao
